# Contributions of Hope in physical activity and exercise goal attainment in college students

**DOI:** 10.3389/fpsyg.2024.1499322

**Published:** 2025-01-10

**Authors:** Corinthian E. B. Blythe, Hayami H. Nishio, Alyssa Wright, Perla Flores, Kevin L. Rand, Kelly M. Naugle

**Affiliations:** ^1^Indiana University Indianapolis, School of Health and Human Sciences, Indianapolis, IN, United States; ^2^Indiana University Indianapolis, School of Science, Indianapolis, IN, United States; ^3^Richard L. Roudebush VA Medical Center, Indianapolis, IN, United States

**Keywords:** hope, optimism, exercise, physical activity, student

## Abstract

**Background:**

College students significantly decrease physical activity (PA) over the course of a four-year degree, increasing the risk for chronic disease. Research shows that psychological constructs impact behavior and goal attainment. However, little is known regarding the effect of psychological variables on PA levels in students. This study examined the association of psychological factors, PA, and exercise goals in college students.

**Methods:**

Students completed two questionnaires within one semester approximately 8 weeks apart. The first (Time 1) included the Adult Hope Scale, Life Orientation Test-Revised, Self-Efficacy to Regulate Exercise, International Physical Activity Questionnaire (IPAQ), and goal assessments. The second (Time 2) included the IPAQ, and scales for goal progress and success. One-hundred seven participants completed both surveys and included an exercise goal.

**Results:**

Predictors of exercise goal attainment included moderate PA at Time 1, goal importance, hope-agency, and the hope-agency by major interaction. Predictors of vigorous PA at Time 2 included hope-agency and moderate PA. The only predictor of moderate PA at Time 2 was moderate PA at Time 1. The only predictor of total PA at Time 2 was moderate PA at Time 1.

**Conclusion:**

Higher hope-agency scores predicted self-reported vigorous PA. We also provide evidence that trait hope influences perceived exercise goal achievement over time.

## 1 Introduction

National physical activity guidelines state that adults should participate in at least 150 min of moderate aerobic activity per week (American College of Sports Medicine, 2018). Alarmingly, over 40% of young adults between the ages of 18 and 24 do not meet these physical activity guidelines (American College of Sports Medicine, 2018). Almost half of young adults in this age range are enrolled in college (U.S. Department of Commerce, Census Bureau, Current Population Survey, 2021). Multiple studies demonstrate high sedentary behavior in college students and that the college years are a time when most young adults see a decrease in physical activity (Sparling and Snow, 2002; Caspersen et al., 2000), contributing to weight gain in this cohort (Pope et al., 2017; Carnethon et al., 2004; Caspersen et al., 2000). For example, one study reported that undergraduates in the United Kingdom spent most of their time in three sedentary categories: studying, screen time, and socializing (Rouse and Biddle, 2010). A study in the United States reported that undergraduate students on average spend 10 h a day in sedentary activities (Peterson et al., 2018). Lastly, in China, youth have recently more than doubled their amount of sedentary time (Zhang et al., 2012), demonstrating a global shift toward physical inactivity.

Failure to acquire or maintain health-promoting behaviors can impede future success and well-being in these young adults (Schulenberg et al., 2003). Furthermore, benefits of physical activity are well known across all demographics (Gropper et al., 2012; Dunsten et al., 2010), including improved quality of life and decreased morbidity and mortality. However, the psychological foundations as to why undergraduate students adopt and maintain physical activity behaviors are still unclear (Anderson and Feldman, 2020; Caspersen et al., 2000; Higgins et al., 2014). Therefore, identifying psychological factors that promote or impede physical activity behavior in this young adult age group is warranted.

Prior research suggests that many psychological factors predict behavior among undergraduate students, including hope (Anderson and Feldman, 2020). Snyder defined hope as “the perceived capability to derive pathways to desired goals and motivate oneself via agency thinking to use those pathways (p. 249)” (Snyder, 2002). The underlying assumption of hope theory is that human actions are goal directed (Snyder, 2002). Pathways refers to the plans individuals create to reach their goals; whereas, agency thinking is the thoughts related to the ability to initiate and continue movement toward the goals. Hope theory posits that agency is a critical factor in achieving goals, especially when alternate paths are required, because it promotes flexibility and creativity to continue pursuing goals while facing adversity (Snyder, 2002). Early research revealed that individuals higher in hope exhibit positive emotions during goal pursuits and cope more effectively with stressors that could impede reaching the goal (Snyder et al., 1991; Snyder, 2002; Tennen et al., 2002).

The goals that people establish range vastly from a simple motor command that happens in seconds to abstract reasoning about life in the future. In a novel study, Feldman and colleagues demonstrated that hope predicts future goal attainment in college students, with goal topics varying widely (Feldman et al., 2009). Hope has since been predictive of behavior in a variety of domains (Snyder et al., 1991; Snyder, 1994), such as athletic performance in Division 1 track athletes (Curry et al., 1997), pain tolerance (Snyder et al., 2005), academic performance in law school (Rand et al., 2011), and grade expectancies in college students (Feldman et al., 2009). Despite the promising area of research on hope and human behavior, little is known regarding hope’s effect on exercise-related goals or physical activity. In one of the rare studies evaluating hope and physical exercise, Anderson and Feldman (2020) revealed that agency, but not pathways, predicted frequency of exercise in adults, even after controlling for exercise-self-efficacy and optimism. However, this study was cross-sectional; therefore, whether hope can predict future exercise behavior remains unknown. Additionally, no research has evaluated whether hope predicts exercise-related goal attainment.

To address this gap in the literature, the purpose of this longitudinal observational study was twofold. First, we tested whether hope predicted perceived exercise goal attainment in undergraduate students. Second, we determined whether hope longitudinally predicted physical activity behavior in these students. We assessed hope, goals, goal importance, physical activity levels, self-efficacy for exercise, and optimism in undergraduate students during the first month of the semester. During the last month of the semester, we assessed perceived goal attainment and physical activity levels. We hypothesized that hope, particularly agency, would predict exercise goal attainment and moderate to vigorous physical activity in undergraduates, even after controlling for relevant demographics and other psychological variables.

## 2 Methods

### 2.1 Participants

Participants included 107 Indiana University-Purdue University Indianapolis (IUPUI) undergraduate students (93 females and 14 males) that were 18 to 43 years of age (*M* = 20.39, SD = 4.28). Inclusion criteria for the study required participants to be enrolled at IUPUI as an undergraduate student. Participants were excluded if they were under 18 years of age. Participants were recruited using two methods. Participants were recruited from the Introduction to Psychology courses at IUPUI. Students in these courses partake in research studies as one means of fulfilling the requirements of their introductory course. Students were also recruited from undergraduate courses offered through the School of Health and Human Science (SHHS), with most of these students enrolled in exercise science majors. Students recruited from SHHS received an in-person scripted verbal explanation of the study, the website link and QR code to participate, along with the researcher’s contact information.

### 2.2 Procedure

Approval to administer the survey and execute this study was provided by the Institutional Review Board from Indiana University. Data collection occurred at two time points, separated by approximately 2 months. The first part of this study occurred within the first 4 weeks of the fall semester. Students were provided a link to the Time 1 questionnaires utilizing the survey tool Qualtrics. Once they opened the link, participants were immediately presented with the informed consent document and were instructed to acknowledge that they read and understood the informed consent document before beginning the questionnaires. Next, participants were prompted to begin the Time 1 Questionnaire, which started by asking about demographic information followed by asking about their personal top five goals they were currently pursuing. Next, they were presented with the goal importance scales, adult hope scale (Snyder et al., 1991), Life orientation test – revised (Scheier et al., 1994), Self-efficacy to regulate exercise scale (Bandura, 2006), and international physical activity questionnaire - short form (Lee et al., 2011). Once they completed the questionnaires, a standardized message thanked them for their participation and reminded them that a researcher would follow up in approximately 8 weeks.

Approximately 8 weeks after students completed the Time 1 questionnaire, they were sent the Time 2 questionnaire email. This email was individualized for each participant to remind them of their unique goals from the responses on the Time 1 questionnaire and a link to complete the Time 2 questionnaires. The Time 2 questionnaires included the International Physical Activity Questionnaire-Short form and assessment of the participants’ perceived level of goal attainment for each goal. Following completion of the Time 2 questionnaire, the participants received a copy of the debriefing form and researcher contact information if they should need any further information or have questions pertaining to the study.

### 2.3 Outcome measures

#### 2.3.1 Demographics

Participants were asked to provide their age, biological sex, college major, standing class, height,

weight, living arrangements, employed working hours, college credits enrollment, grade point.

average, and marital status.

#### 2.3.2 Goals survey and goal importance

At Time 1, students were asked to list their top five goals with the following question, “Please provide a short description of the 5 most important goals that you are currently pursuing.” Participants rated the importance of each goal on a 0 to 10 numeric rating scale, with 0 indicating not at all important and 10 indicating extremely important (Feldman et al., 2009).

#### 2.3.3 Goal attainment

At Time 2, participants were asked the following two questions to assess goal attainment (Feldman et al., 2009):

1. “Indicate what percentage of progress you made toward achieving this goal.” Participants selected from a range of 0 to 100%.
2. “How successful were you with each goal?” Participants rated their success on a scale of 0 (not successful at all) to 6 (very successful).

Similar to the Feldman et al. (2009) study, the values obtained for each question were standardized and then summed to yield on overall goal attainment index for statistical analysis.

#### 2.3.4 Adult hope scale

AHS is a measure of trait hope in adults formed from two subscales measuring agency and pathways thinking (Snyder et al., 1991). The AHS includes 12 statements that participants rate from 1 (definitely false) to 8 (definitely true) for how the statements describe them. Of the 12 items, four measure agency (e.g., I energetically pursue my goals), four measure pathways (e.g., “I can think of many ways in life to get the things that are most important to me”), and four are distractors that are not included in the final summation. The AHS has shown good convergent and divergent validity. Total scores range from 8 to 64.

#### 2.3.5 Life orientation test-revised

The LOT-R is a ten-question scale measuring trait optimism (Scheier et al., 1994). The LOT-R asks participants to rate on a 5-point scale from 0 (strongly disagree) to 4 (strongly agree) to what extent they agree with 10 statements. (e.g., “Overall, I expect more good things to happen to me than bad”). The LOT-R has been tested for accuracy and has good validity (Chiesi et al., 2013). Total scores range from 0 to 40.

#### 2.3.6 International physical activity questionnaire-short form

The IPAQ is a measure of physical activity that asks subjects to recall the amount of time during the past week spent on vigorous activity, moderate-intensity activity and walking (e.g., “During the last 7 days, on how many days did you walk at least 10 min?”) (Lee et al., 2011). Scores were calculated in terms of MET-minutes/week for vigorous activity, moderate activity, walking, and Total activity. The IPAQ-SF has shown acceptable concurrent and construct validity and test–retest reliability (0.66–0.89) (Craig et al., 2003; Lee et al., 2011).

#### 2.3.7 Self-efficacy to regulate exercise scale

This questionnaire measures individual beliefs about their ability to perform and overcome barriers to exercise on a 100-point scale that captures emotional issues and external factors (Bandura, 2006; e.g., “how confident are you that you could exercise when in a bad mood?”). This scale has been tested and found valid (Bandura, 2006). Total scores range from 0 to 1,800.

### 2.4 Statistical analysis

For a power of 0.80 with alpha set at 0.05 and a moderate effect size of *f*^2^ = 0.15, a power calculation revealed that 92 participants would be needed to adequately predict goal progress and success with up to five predictor variables.

Not all participants provided an exercise-related goal. The current study focused specifically on predicting exercise-related goal attainment, thus only participants who provided an exercise-related goal were included in the data analysis. Descriptive statistics were conducted for the whole sample (which provided an exercise goal) and each subgroup defined by exercise related major vs. non-exercise science related major. The separation between exercise-related majors and non-exercise-related majors was because students in exercise-related majors likely have had more education on exercise principles, and potentially greater exercise self-efficacy and PA levels. Shapiro–Wilk’s test of normality was used to determine the normality of the distribution of the outcome measures. All of the primary outcome measures, except for Self-efficacy to Regulate Exercise Scale, were distributed non-normally. Data were presented as frequency for sex, race, and class, and mean ± standard deviation (SD) or median (interquartile range) depending on the distribution of the data. Mann–Whitney U tests were used to determine whether non-normally distributed outcome variables differed by major (exercise vs. non-exercise), and independent t-tests were used to determine whether normally distributed outcome variables differed by major.

Bivariate Spearman rank correlations were conducted to determine the relationships between goal attainment and the IPAQ variables (moderate PA, vigorous PA, total PA) at Time 2 with personal variables (e.g., age, BMI), and Time 1 variables of IPAQ-vigorous PA, IPAQ-moderate PA, IPAQ-total PA, AHS-agency, AHS-pathways, LOT-R, EXSE, and goal importance. Next, separate multivariate linear regression analyses were conducted to determine predictors of goal attainment, vigorous PA, moderate PA, and total PA at Time 2. Variables chosen based on previous research that were significantly correlated with the dependent variables were initially entered into the regressions. Only significant predictors were retained in the final models. We also explored whether the interaction between major (exercise science related vs. non-exercise science related) and AHS-agency, and major and AHS-pathways affected the results of the models. All statistics were conducted using IBM SPSS Statistics version 27. The *p*-value for significance was set at *p* < 0.05.

## 3 Results

### 3.1 Participant characteristics

The participants in this study were undergraduate students enrolled at IUPUI. Two-hundred and seventy-one students enrolled in the study, and 107 were retained for organically choosing an exercise goal and completing the entire study. Detailed characteristics of the whole sample (*n* = 107) and the sample divided into exercise related majors (*n* = 53) and non-exercise related majors (*n* = 54) are reported in Table 1. The students were mostly White, female, and between the ages of 18–43 (84.1% were between 18 and 21 years of age). A Mann–Whitney U test indicated that exercise-science majors had significantly greater LOTR scores (optimism) compared to non-exercise science majors, *p* = 0.022. Also, an independent t-test indicated that exercise-science majors had significantly greater self-efficacy to regulate exercise compared to non-exercise science majors, *p* = 0.031. No other variables differed between exercise-science and non-exercise science related majors (*p*’s > 0.05). Table 1 displays the demographic outcomes of the participants.

**Table 1:** Sample demographics and characteristics.

Variable	Sample (*n* = 107)	Non-ex major (*n* = 54)	Ex major (*n* = 53)
Age (y)	20.4 ± 4.3	21.1 ± 5.4	19.7 ± 2.5
BMI T1, kg/m^2^	26.1 ± 5.9	26.9 ± 6.9	25.1 ± 4.3
Sex, % female	93 (86.9%)	49 (90.7%)	44 (83%)
Class			
Freshman	49 (45.8%)	28 (51.9%)	21 (39.6%)
Sophomore	27 (25.2%)	15 (27.8)	12 (22.6%)
Junior	19 (17.8%)	3 (5%)	16 (30.2%)
Senior	8 (7.5%)	4 (7.4%)	4 (7.5%)
Other	4 (3.7%)	4 (7.4%)	0 (0%)
IPAQ, met-min/wk Median [Q1, Q3]			
Walking	1,386 [462, 2,772]	1,584 [396, 4,307]	1,386 [578, 2,772]
Moderate	720 [0, 2,400]	720 [0, 2,400]	720 [240, 2,400]
Vigorous	960 [0, 2,880]	480 [0, 1,500]	1920 [480, 3,600]
Total	4,884 [1829, 9,264]	4,610 [1,503, 10,759]	4,884 [2,380, 8,919]
Self-efficacy to regulate exercise (0–1,800)	1,005 ± 375	922 ± 420.7	1085.7 ± 308.6
Optimism (0–40)	25 [21, 29]	23 [18, 28]	26 [23, 29]
Goal Importance (0–10)	9 [7, 10]	8 [7, 10]	9 [8, 10]
Hope			
Agency (4–32)	26 [23, 29]	26 [22.75, 29]	27 [23, 29]
Pathways (4–32)	25 [23, 27]	24.5 [22, 26.25]	26 [23, 28.5]
Total (8–64)	51 [46, 56]	50 [46, 55]	51 [46.5, 56.5]
Goal attainment, z score	0.28 [−1.60, 1.43]	0.08 [−1.93, 1.39]	0.63 [−1.41, 1.46]

Values denote means ± standard deviation, n (percentage of n), or median [interquartile range].

### 3.2 Correlational analyses

Spearman correlation coefficients are presented in Table 2. Goal attainment was significantly and positively correlated with several variables at Time 1, including moderate PA, vigorous PA, Total PA, exercise self-efficacy, optimism, goal importance, and hope agency. Goal attainment was also negatively correlated with BMI (*r* = −0.25, *p* = 0.017). Total PA at Time 2 was significantly and positively associated with all of the Time 1 PA variables, having the strongest relationships with moderate PA and total PA. Vigorous PA at Time 2 was positively associated with moderate PA, vigorous PA, Total PA, exercise self-efficacy, goal importance, and hope-agency at Time 1. Moderate PA at Time 2 was only significantly and positively associated with moderate PA, total PA, and exercise self-efficacy at Time 1.

**Table 2:** Correlation coefficients for the association of goal attainment and PA variables at Time 2 with psychological variables and PA variables at Time 1.

Variable	Goal attainment	Total PA Time 2	Vigorous PA Time 2	Moderate PA Time 2
IPAQ Time 1				
Walking	−0.019	0.237*	0.065	0.099
Moderate	0.303**	0.468**	0.415**	0.376**
Vigorous	0.323**	0.268**	0.381**	0.099
Total	0.238*	0.457**	0.339**	0.273**
Exercise self-efficacy	0.287**	0.169	0.394**	0.391**
Optimism	0.218*	0.068	0.167	0.111
Goal Importance	0.222*	0.139	0.195*	0.049
Hope				
Agency	0.224*	0.105	0.193*	0.057
Pathways	0.045	−0.026	0.077	0.127
Total	0.164	0.048	0.163	0.098

**Correlation is significant at the 0.01 level (2-tailed). *Correlation is significant at the 0.05 level (2-tailed). PA, Physical activity; IPAQ, International Physical Activity Questionnaire.

### 3.3 Multivariate linear regression analyses

#### 3.3.1 Hypothesis 1: hope at the beginning of the semester will predict exercise goal attainment near the end of the semester

**Figure 1:**
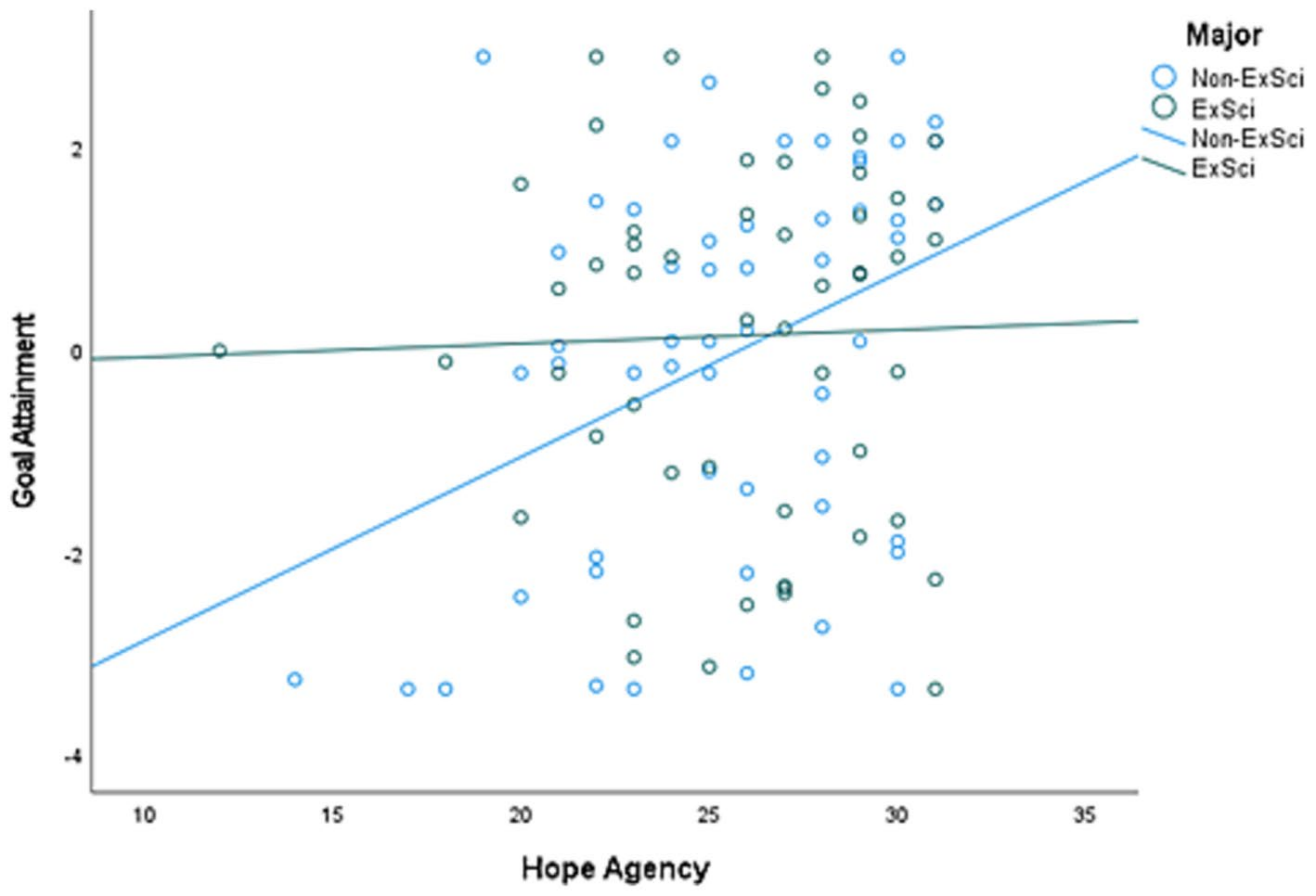
A scatterplot graph depicting the interaction of major and hope-agency and the impact of goal attainment. Students in a non-exercise related major (Non-ExSci) demonstrated a stronger relationship of hope-agency and goal attainment compared to students in an exercise-related major (ExSci).

Potential predictors were chosen from the variables significantly correlated with goal attainment. We also explored the interaction terms of major (exercise vs. non-exercise) and the psychological variables as predictors (e.g., major and hope-agency, major and exercise self-efficacy). To create the interaction term, the psychological variables were first standardized as a z-score and then multiplied with major. The final regression model included only significant variables, other than keeping major as a predictor. The final model was significant (*p* < 0.001) with an R^2^ of 0.215. Significant predictors of goal attainment included moderate PA at Time 1, goal importance, hope-agency, and the hope-agency by major interaction term. See Table 3 for results of the regression analysis. The relationship between hope-agency and goal attainment was stronger for those in non-exercise-related majors compared to exercise-related majors as shown in Figure 1.

**Table 3:** Multivariate linear regression analyses predicting goal attainment (*n* = 107).

	B	Std. Error	Beta	*t*	Sig.
Major	0.125	0.334	0.034	0.373	0.710
Goal importance	0.220	0.095	0.216	2.309	0.023
Moderate physical activity	0.000	0.000	0.252	2.749	0.007
Hope agency	0.710	0.208	0.407	3.417	0.001
Agency by major interaction	−0.910	0.318	−0.343	−2.864	0.005

#### 3.3.2 Hypothesis 2: hope at the beginning of the semester will predict physical activity near the end of the semester

Potential predictors were chosen from the variables significantly correlated with each PA variable at Time 2. We also explored the interaction terms of major (exercise vs. non-exercise) and the psychological variables as predictors (e.g., major and hope-agency, major and exercise self-efficacy), but the interaction terms were not significant predictors of any of the PA variables. The final regression models included only significant variables. The final model for moderate PA was significant (*p* = 0.002), with an R^2^ of 0.153. The only significant predictor was moderate PA at Time 1. The final model for vigorous PA was also significant (*p* < 0.001), with an R^2^ of 0.208. Significant predictors included moderate PA at Time 1 and hope-agency. Thus, those with higher levels of moderate PA and hope-agency at the beginning of the semester reported higher levels of vigorous PA at the end of the semester. Lastly, the final model for total PA was significant (*p* < 0.001) with an *R*^2^ of 0.247. The only significant predictor was moderate PA at time 1 (Table 4).

**Table 4:** Multiple regression analyses for PA variables at Time 2 (*n* = 107).

	B	Std. Error	Beta	t Sig.	
Vigorous PA Time 2					
Hope agency	562.895	246.7	0.209	2.282	0.025
Moderate PA	0.528	0.132	0.375	3.987	<0.001
Moderate PA Time 2					
Moderate PA	0.494	0.120	0.405	4.123	<0.001
Total PA Time 2					
Moderate PA	1.378	0.258	0.494	5.343	<0.001

PA, Physical activity.

## 4 Discussion

This longitudinal study tested hope as a predictor of perceived exercise goal attainment and self-reported physical activity in college-students. Several key findings emerged from the data. First, hope at the beginning of the semester, specifically the agency subscale, predicted perceived exercise goal attainment near the end of the semester. Second, hope-agency and previous physical activity predicted self-reported vigorous physical activity levels at the end of the semester. These findings bring science closer to understanding the role of hope in physical activity and exercise goal pursuit.

### 4.1 AHS prediction of perceived exercise goal attainment

The results partially supported our hypothesis that hope measured at the beginning of the semester would predict perceived exercise goal attainment at the end of the semester. Specifically, the subscale hope-agency, but not hope-pathways, at the beginning of the semester predicted perceived exercise goal attainment at the end of the semester. This finding provides additional evidence for the benefit of parsing out measures of hope into pathways and agency subscales when measuring its influence on behavior. Hope-agency was a significant predictor of exercise goal attainment even when controlling for goal importance and previous physical activity levels. Prior research has also reported that hope-agency versus hope-pathways is a more reliable predictor of goal attainment in college students (Feldman et al., 2009; Anderson and Feldman, 2020). These results suggest that determination to reach a goal (agency) may exert greater influence on perceived goal success than identifying pathways to achieving the goal. One theory as to why hope-pathways exerts little influence on goal-attainment is the short time span allocated in these studies (Feldman et al., 2009). Time may be a critical construct for generating multiple pathways to reaching an exercise goal (Feldman et al., 2009). For example, running a half-marathon, preparing for cross-fit games, or competing in a body-building competition takes many weeks, months, or years to prepare. Future research should investigate whether hope-pathways plays a larger role in goal attainment for goals with a longer duration for achievement.

Interestingly, our results also revealed that the relationship between hope-agency and perceived exercise goal attainment was stronger in non-exercise related major students compared to exercise related major students (Figure 1). Several reasons could explain why the relationship between hope and exercise goal attainment differed between these two groups of students. The exercise students, with exposure to exercise science courses, likely had greater knowledge of exercise principles and benefits. Additionally, those who pursue an exercise-related degree may have developed a deeper appreciation for exercise habits before college compared to those who chose a major not associated with exercise. Indeed, one study identified the top three reasons students pursue an exercise science – related degree to include interest, job opportunities, and aptitude (Vaartstra et al., 2017). In the current study, the exercise science students also demonstrated higher self-efficacy to regulate exercise and optimism compared to the non-exercise science students. Prior studies have demonstrated a positive relationship between physical activity and optimism (Kavussanu and McAuley, 1995; Pavey et al., 2015). Thus, interest and aptitude along with education and exposure to exercise-related courses may have washed out the effect of hope on exercise goal attainment in exercise science students. On the other hand, non-exercise majors may require more agentic cognitions to achieve their exercise goals. Further research is needed to understand what psychological traits impact exercise behaviors broadly in different career trajectories.

While not the focus of the study, we also found that goal importance and self-reported moderate physical activity at the beginning of the semester predicted perceived exercise goal-attainment. Thus, students who rated their goal as more important and reported more moderate physical activity were more likely to perceive goal attainment at the end of the semester. Prior studies have shown goal importance to impact goal attainment (Feldman et al., 2009) and post-education vocational success (Nurmi et al., 2002). Furthermore, our results are in accordance with multiple studies demonstrating that engaging in PA is a good predictor of staying physically active later in life (Smith et al., 2015; Itoh et al., 2017).

Additionally, our results indicated that optimism, another foundational concept of positive psychology, did not predict exercise goal attainment. Hope and optimism have been used interchangeably in past literature despite being two distinct constructs and delineating these constructs provides further insight to goal-directed behavior (Tennen et al., 2002; Feldman et al., 2009). Dispositional optimism refers to general positive outcome expectations (Carver and Scheier, 1998); whereas, hope is the perceived ability to procure pathways and maintain agency to achieve a goal (Snyder, 2002). Hope may have a direct effect on goal attainment, whereas optimism has an indirect effect. Past studies analyzing optimism, but not hope, have shown an indirect effect of optimism on goal progress (Monzani et al., 2015) and therapeutic attendance to assist academic goals (Geers et al., 2010). A consideration may be those with higher measures of dispositional optimism are more likely to replace physical activity goals when faced with limitations to maintain psychological well-being (Duke et al., 2002). Optimism has also been shown to exude a greater impact on goal expectancy, compared to hope, when participants perceive the goal outcome as out of their control (Shanahan et al., 2020). Students who perceive their exercise goal as outside their control (i.e., the belief that genetics determine fitness level, body composition) or encounter stressors (i.e., new environment, new peers, exams) may rely on optimistic cognitions to passively cope with failure to achieve their goal (Rand et al., 2011; Shanahan et al., 2020).

Lastly, self-efficacy to regulate exercise did not predict goal attainment or PA, which is in line with results found by Anderson and Feldman (2020). We found that self-efficacy to regulate exercise was significantly correlated with goal attainment and PA, but was not a significant predictor once hope, goal importance, and previous PA were accounted for. However, studies solely examining exercise self-efficacy show interventions which increase exercise self-efficacy increase energy expenditure in sedentary populations (Buckley, 2014). Notably, a key difference between self-efficacy and hope is that self-efficacy involves the belief that one is capable of a behavior; whereas hope involves commitment to engage in the behavior. Future research should continue to investigate the role of exercise self-efficacy in initiating and maintaining physical activity.

### 4.2 AHS prediction of physical activity

We hypothesized that hope at the beginning of the semester would predict physical activity near the end of the semester, and the data mostly supported our hypothesis. Hope-agency measured at the beginning of the semester predicted vigorous physical activity near the end of the semester, even after controlling for physical activity at the beginning of the semester. These results are similar to the Anderson and Feldman (2020) study that reported goal-specific hope correlated with the frequency of moderate and strenuous exercise. Interestingly, Anderson and Feldman observed that goal specific hope, but not trait hope (i.e., the measure used in the current study), predicted frequency of exercise. Several differences between the current study and the Anderson study could explain these contrasting findings regarding trait hope’s prediction of physical activity/exercise. First, the focus of our study was the volume of physical activity rather than the frequency of exercise as in the Anderson study. Past literature has used physical activity and exercise synonymously; however, they are distinct and nuanced constructs (Caspersen et al., 1985). Both exercise and physical activity include bodily movement using skeletal muscle, energy expenditure, and correlate with physical fitness. Exercise differentiates from physical activity in regard to planning, structure, and objective goals to maintain or improve physical capabilities (Caspersen et al., 1985). Measuring exercise may only capture a small portion of time when an individual engages in deliberate, structured, and outcome-based movement compared to measuring physical activity which captures the waking hours of an individual and how much they move throughout their waking time. Though similar, these distinctions could explain why goal-specific hope, but not trait hope, was significant in predicting exercise in their study. Another factor that may have impacted different contributions of hope is that Anderson and Feldman used a cross-sectional study design, whereas, our study was longitudinal. Thus, our study focused on predicting future physical activity rather than current exercise or physical activity. Additionally, we did not measure goal-specific hope. Future research should investigate whether goal-specific hope would be a stronger predictor of perceived exercise goal attainment and physical activity compared to trait hope.

Importantly, our study along with Anderson and Feldman (2020) reveal that hope-agency is a stronger predictor of vigorous/strenuous (respectively) physical activity compared to lower intensities. The importance of PA is exceptionally well documented, and vigorous PA contributes to increased health and well-being. Studies have shown that compared to light PA, vigorous PA offers greater reduction in mortality risk (Saint-Maurice et al., 2018; Gebel et al., 2015), reduced risk of type 2 diabetes (Hu et al., 1999), and greater improvements in physical impairments (Kaleth et al., 2013). Future research needs to determine whether the construct of hope can be utilized to facilitate college students meeting the physical activity guidelines of at least 150 min of moderate aerobic activity per week or 60 min of vigorous PA between 3 days per week.

Meeting the guidelines for PA typically begins with conscience intentions to be active, especially for students. Nothwehr et al. (2013) analyzed diet and physical activity-related behavioral strategies in a cross-sectional sample of adults in the Midwest region of the United States. They did not quantify physical activity, but instead captured intentions to be physically active and have physically active social interactions. Similar to our results, Nothwehr et al. (2013) demonstrated that hope-agency positively correlated with self-monitoring of physical activity. However, they also reported correlations with self-monitoring of physical activity and total hope, as well as self-monitoring of physical activity and hope-pathways. Though not surprising, the difference in recalling previous physical activity in the context of walking, moderate, and vigorous physical activity compared to reflecting on how one self-monitors PA may involve deeper processing of pathways thinking.

### 4.3 Hope theory

Our study expands the evidence in support of Hope Theory and adds further understanding about achieving goals related to exercise. There may be an emerging, and potentially evolving piece of Hope Theory, where agency is the primary driver of goal achievement, compared to pathway formation. Most people, especially students, have unlimited access to instantaneous information as a result of innovation in technology. Students may implicitly acknowledge the numerous paths to achieve physical activity and exercise goals and may place a greater emphasis (or lack of) on remaining dedicated to achieving their goals. More research is needed to understand how hope impacts goal-directed behavior as technology and the availability of information rapidly evolve.

### 4.4 Limitations

Our study provides novel insights to psychological factors that impact physical activity and exercise goal achievement in a collegiate setting. However, several limitations of this study should be acknowledged. First, while our data has notable implications for college students, these results may not apply to other populations, such as older adults or children. The second limitation is our study unexpectedly measured mostly female participants (86.9%). This predominantly female sample may restrict the generalizability of our findings to male populations. Third, it is also possible that a portion of the exercise science participants were student-athletes, although we did not collect this information. Student-athletes have a combination of education and sports requirements. Prior research indicates that the student-athlete identity of college-aged student-athletes is pronounced (Lupo et al., 2017a), with greater motivation to succeed in the dual student-athlete role (Lupo et al., 2017b). Thus, college student-athletes would potentially be required and/or motivated to reach exercise-related goals as part of their athletic team participation. Future exercise-related research in college students should assess athletic sport team participation to allow for further sub-group analysis of the relationship between psychological factors and exercise. The fourth limitation is that the IPAQ questionnaire is a retrospective self-report of physical activity inquiring the participant about the previous 7 days. Self-report requires participants to accurately recall information and avoid modifying their responses to appear favorable. Recall introduces an honest bias of potentially retrieving incorrect responses to the amount or intensity of physical activity. Additionally, we asked participants to subjectively gage how much progress and success they made in approximately 8 weeks toward goal attainment, which may not reflect actual goal attainment. Previous research has shown that people modify goals based on perceived attainability, internal resources, external resources, age, and coping with deficits (Duke et al., 2002). Subjective reporting of perceived goal attainment could be artificially inflated as a protective psychological mechanism to maintain participant congruence. It is possible that having higher hope leads one to overestimate achievement toward goals. Future studies should consider both, objectively and subjectively quantifying goal attainment to avoid mono-method bias. Finally, prior research indicates an increasing level of perceived stress, anxiety, and depression among college students (Brown, 2018). As physical and mental health are intrinsically related, future research should also investigate the role of these mental health factors on physical activity levels and exercise goal attainment in college students.

### 4.5 Conclusion

In conclusion, our longitudinal study demonstrated that higher hope scores predicted self-reported vigorous physical activity approximately 8 weeks later. We also provided the first evidence that trait hope influences perceived exercise goal achievement over time. Perceived exercise goal importance and feeling a sense of agency to achieve the goal predicted goal achievement. Future research should explore why developing pathways was not predictive of goal achievement in our study. Reaching a goal has been conceptualized as identifying the ways to achieve it and the will to do so. However, pathways was not a strong driver, like agency, in our sample. Lastly, we provided additional evidence that previous physical activity levels are still a strong predictor of future physical activity. Which begs the question, what sets a sedentary person into motion to pursue and achieve a healthy state of physical fitness?

## Data Availability

The raw data supporting the conclusions of this article will be made available by the authors, without undue reservation.
